# Insights into udder health and intramammary antibiotic usage on Irish dairy farms during 2003-2010

**DOI:** 10.1186/2046-0481-65-7

**Published:** 2012-03-28

**Authors:** Simon J More, Tracy A Clegg, Luke O'Grady

**Affiliations:** 1 Centre for Veterinary Epidemiology and Risk Analysis, University College Dublin, Belfield Dublin 4, Ireland; 2 Herd and Veterinary Public Health, UCD School of Agriculture, Food Science and Veterinary Medicine, University College Dublin, Belfield Dublin 4, Ireland

## Abstract

By international standards, Ireland is a relatively small dairy producer. However, the industry plays a critical role to the national economy, accounting for approximately 3% of national gross domestic product. This paper presents insights into udder health and intramammary antibiotic usage on Irish dairy farms during 2003-2010, based on data from several sources. Three data sources were used, including data on milk recording data, intramammary antibiotic sales and animal health assessment. The milk recording data included a single unadjusted herd-level somatic cell count (SCC) value for each herd at each milk recording, being the arithmetic mean of cow-level SCC of each cow at that recording, weighted by cow-level yield. These data were used to calculate the percentage of herds each month where the unadjusted herd SCC exceeded 200,000 and 400,000 cells/mL. Two logistic generalised estimating-equations (GEE) models were developed, the outcome variable being either the probability that the monthly SCC of a herd was greater than 400,000 cells/mL or less than or equal to 200,000 cells/mL. Spring herds had a lower probability of a high SCC (> 400,000 cells/mL) during February to October compared to non-Spring herds but a higher probability between November to January. The odds of a high SCC were greater in 2005, 2006, 2009 and 2010 but less in 2007 and 2008 compared to 2004. Smaller herds had higher odds of having a high SCC compared to larger herds. We present the number of intramammary tubes and the quantity of active substance (kg) sold annually in Ireland during 2003-2010. We infer an incidence of clinical mastitis of 54.0 cases per 100 cow-years at risk, assuming 4 tubes per treatment regime, one affected quarter per cow, tubes restricted to clinical cases only and 100% of treated cases considered new cases, based on data collected on sales of in-lactation intra-mammary antibiotics. With differing assumptions, this estimate varied between 25.8 and 77.0 cases per 100 cow-years at risk. Using data on sales of dry cow therapy intra-mammary antibiotics, we also infer that most Irish dairy farmers use blanket dry cow therapy. It is important that Ireland has an objective understanding of current levels of udder health, to facilitate benchmarking and improvement into the future. Udder health is a concern on a number of Irish dairy farms. High SCC results were present throughout the year, but more marked towards the start and end of each milking season. Animal Health Ireland recently commenced a major national programme, *CellCheck*, in collaboration with a broad range of stakeholders, to support national SCC improvement. In this paper, relevant European and national legislation is also reviewed.

## Introduction

By international standards, Ireland is a relatively small dairy producer [1]. During 2003 to 2007, the number of approved dairy producers fell from 26,883 to 20,182, whereas the total volume of milk collected remained relatively steady. In 2008, the Irish dairy industry produced 5.1 million tonnes of milk, equivalent to 0.88% of global production [2]. However, the industry plays a critical role to the national economy, accounting for approximately 3% of national gross domestic product [3]. Approximately 85% of annual production is exported.

The quality of Irish milk and other dairy products is of paramount importance, both to domestic and international consumers. Ireland is the world's leading producer of infant nutrition products, producing 15% of the world's powdered infant formula [3]. 'In 2009, the infant milk formula market was valued at approximately US$10 billion, growing on average at 15% each year [4]. Ireland is seeking a 50% increase in milk production by 2020, using 2007-09 as a baseline [5].

A broad range of criteria is used to assess the quality of raw milk, relating to composition (butterfat, crude protein, lactose, milk solids etc.) and hygiene (total bacterial count, somatic cell count, residues of veterinary medicines including antibiotics). Somatic cell count (SCC) is the most important, single indicator of milk quality, reflecting the health status of the mammary gland (so-called 'udder health') and the risk of non-physiological changes to milk composition [6].

There are substantial costs associated with sub-optimal udder health, both on-farm and during processing, as reviewed previously [1]. Herds with udder health problems, generally as a consequence of mastitis (inflammation of the mammary gland), are also at increasing risk of antibiotic residue violation, as a result of increased antibiotic usage [7,8]. SCC underpins national and international regulation for milk quality [8], and standards for total bacterial counts (TBC), SCC and residues of veterinary medicines are each defined within EU legislation (see Additional file 1). Uninfected quarters have an average SCC of approximately 70,000 [9,10] to 100,000 [11] cells/mL, although this does increase with age. SCC is also influenced by stage of lactation as a consequence of dilution, leading to increases in SCC with reduced milk volume [12,13]. An elevated SCC is indicative of mastitis, generally caused by presence of infectious microorganisms [6]. Non-infected and infected quarters are generally distinguished using a cut-off of 200,000 cells/mL, with a sensitivity and specificity of 74.5% and 89.6%, respectively [14]. The relationship between infection and SCC is closest at the level of the quarter, however, SCC is also a proxy for udder health at the level of the cow, the herd and the broader population [10]. At the herd level, where a threshold of 200,000 cells/mL is considered a useful predictor of intramammary infection [15,16], longitudinal data (collected over time) is needed to monitor SCC [10].

There have been few publications on udder health and intramammary antibiotic usage on Irish dairy farms. Nonetheless, some information from earlier work [17-19] is available. The current paper seeks to build on this earlier work, presenting insights into udder health and intramammary antibiotic usage in the Irish dairy industry during 2003-2010, based on data from several sources.

## Materials and methods

### The data

Three data sources were used in this study, as follows:

#### Milk recording data

Milk recording is conducted voluntarily in Ireland, with all data being managed by the Irish Cattle Breeding Federation (ICBF). We obtained data about all milk recordings conducted in Ireland during 2003 to 2010, inclusive. The data included a single herd-level SCC value for each herd at each milk recording, being the arithmetic mean of cow-level SCC of each cow at that recording, weighted by cow-level yield. In addition, at each milk recording, data were available on the number of cows sampled and the total volume of milk recorded. For each herd each year (all years except 2010, where these data were not available), we also obtained the percentage of calves born between 01 January to 30 June (considered the spring-calving period). In this dataset, the reference population was all milk recording herds in Ireland, and the herd-level SCC in each herd at each milk recording was the primary unit of interest.

#### Intramammary antibiotic sales data

GfK Kynetec, an international market research company specialising in agriculture and animal health, gather data on all intramammary sales conducted through each of the five main veterinary wholesalers in Ireland. According to GfK Kynetec, this is likely to represent an estimated 85% of all sales of these products in Ireland. We obtained data from GfK Kynetec, summarised yearly for each year during 2003 to 2010 inclusive, of sales of intramammary antibiotic products for cows during lactation and at drying off. In this dataset, the reference population was all dairy herds in Ireland, and defined quantities of intramammary antibiotic product (either g of active ingredient or number of mastitis tubes) were the units of interest.

#### Animal health assessment data

In Ireland, each dairy farm is inspected periodically (generally annually) by a veterinarian to certify compliance with animal health requirements (the Animal Health Inspection of Dairy Cows or 'dairy cert.'). Further information about the dairy cert. is presented in Additional file 1. We obtained a yearly summary from the national Department of Agriculture, Food and the Marine (DAFM) of the number of herds non-compliant with the dairy cert., during the years 2003 to 2009 (at the time of writing, no data were available for 2010). In this dataset, the reference population was all dairy herds in Ireland, and the non-compliant dairy farm was the unit of interest.

### Data analysis

#### Milk recording data

Preliminary screening of the milk recording data was conducted to identify and remove all duplicate (non-valid) records (arbitrarily, duplicate on-farm milk recordings less than 21 days apart). Recordings with more than 50% of samples unreadable, as were milk recording data with less than 10 cows and/or a SCC less than 20,000 cells/mL. We considered these latter circumstances unrepresentative of the national herd.

We calculated the number of valid milk recordings (in total and by year), the number of milk-recorded herds (herds with at least one valid milk recording during a calendar year; in total and by year) and the number of milk recordings per herd (by year). Herd size was calculated, based on the median number of cows per herd across all milk recordings for that herd. We identified a subset of herds as 'strictly spring-calving' (all herds with 100% of calves born during the spring-calving period). In 2010, herds were allocated based on 2009 data.

We calculated the unadjusted herd SCC (median and quartile range) per month (the monthly SCC) in all milk-recorded herds, the percentage of herds each month where the unadjusted herd SCC exceeded 200,000 and 400,000 cells/mL, and the geometric mean every 6 months, either January to June or July to December. In each case, if more than one valid milk recording was available for any month, only the first was used. Data manipulation and analyses were conducted using SAS version 9.1.3 (SAS Institute, Cary, NC, USA). Graphs were created using Microsoft Excel 2007 (Microsoft Corporation, Redmond, WA, USA).

Two logistic generalised estimating-equation (GEE) models were developed, the outcome variable being either the probability that the monthly SCC of a herd was greater than 400,000 cells/mL or less than or equal to 200,000 cells/mL. In each model, an autoregressive correlation was used to account for the serial correlation between monthly measurements within the same herd. The terms month, year, herd size and Spring calving (Spring = 100% of calves born between January and June, Non-Spring = all other herds) were included in each model as categorical variables. Herd size was based on the maximum number of cows tested within a year and categorised into 4 groups based on the quartiles of the herd size distribution within the respective year. Similarly Spring calving was based on the percentage of calves born between January and June within each respective year. Terms were assessed for inclusion within each model on the basis of the generalised score test. Consistent estimates of coefficient standard errors were obtained using the empirical covariance matrix of parameter estimates resulting from the GEE method. The models were fitted using the SAS GENMOD procedure. Since not all herds were recorded every month, there will be intermittent missing values for some herds. The GENMOD procedure estimates the working correlation from data containing missing values using the 'all available pairs' method, in which all non-missing pairs of data are used in the moment estimators of the working correlation parameters. Data for 2003 was excluded from each model as milk recording data from that year were limited. The fit of the final model was checked using a half-normal plot of the Pearson residuals with a simulated envelope [20].

#### Intramammary antibiotic sales

Each intramammary antibiotic product was categorised by time of application (in-lactation, dry cow) and the antibiotic group(s) of the active substance(s), as follows:

• *Aminocoumarins *(novobiocin);

• *Aminoglycosides *(framycetin, kanamycin, neomycin, streptomycin);

• *Cephalosporins*, either 1st generation (cefacetrile, cefalexin, cefapirin, cephalonium), 3rd generation (cefoperazone), 4th generation (cefquinome);

• *Lincosamides *(lincomycin, pirlimycin);

• *Macrolides *(erythromycin);

• *Penicillins*, either narrow spectrum (β-lactamase sensitive: benzylpenicillin, penethamate; penicillinase-resistance: cloxacillin, nafcillin), moderate spectrum (ampicillin), broad spectrum (amoxicillin and clavulinic acid);

• *Sulphonamides *(sulphadiazine, trimethoprim); and

• *Tetracyclines *(oxytetracycline).

Using Microsoft Excel 2007 (Microsoft Corporation, Redmond, WA, USA), the data were summarised according to the number of tubes sold by time of application, and the quantity of active substance sold by antibiotic group and time of application. These data were then used to estimate:

• *Dry cow therapy (DCT) coverage *(the % of lactating cows receiving dry cow intramammary antibiotic therapy at drying off), after considering the number of lactating dairy cows and the total number of DCT tubes sold.

• *Incidence of clinical mastitis *(number of clinical mastitis cases per 100 cows per year), after considering the number of lactating dairy cows, the number of cows at risk of clinical mastitis, the days at risk per cow, the days after treatment before a subsequent treatment is considered a new case, the number of in-lactation tubes sold, the number of tubes used per treatment regime, the number of affected quarters per cow and the relative use of in-lactation tubes for clinical and subclinical mastitis cases. The incidence rate was calculated based on guidelines published by the International Dairy Federation [21].

Relevant national and EU legislation is presented in Additional file 1.

#### Animal health assessment data

A yearly summary was produced, using available data.

## Results

### Somatic cell counts in milk recording herds

During 2003 to 2010, 297,652 milk recordings were available, including 295,286 valid milk recordings conducted in 9,057 herds. The number of herds by number of milk recordings in each herd per year is presented in Table 1 noting that approximately half of the herds made between 4 and 7 recordings each year. The average (median) herd size was 66.3 (58.0) cows, with a minimum of 10, a maximum of 765 and an interquartile range of between 44 to 78 cows. Table 2 presents summary statistics for herds that milk recorded in 2010. Between 41.4% (in 2008) and 46.3% (2010) of milk recording herds were strictly spring-calving. In 2009, the distribution of herds according to a spring-calving pattern was: 45.1% of herds with 100% spring-born calves, 63.5% with > 95% spring-born calves, 70.0% with > 90% spring-born calves, and 94.1% with > 50% spring-born calves.

**Table 1:** Number of herds by number of milk recordings in each herd per year during 2003 to 2010

Number of milk recordings	2003 (n = 3,296)	2004 (n = 6,287)	2005 (n = 6,033)	2006 (n = 6,228)	2007 (n = 6,230)	2008 (n = 6,400)	2009 (n = 5,776)	2010 (n = 5,924)	Total
1	640	101	128	98	106	130	149	105	1,457
2	815	92	66	94	93	160	141	129	1,590
3	688	150	142	245	246	373	351	333	2,528
4	379	489	738	1,191	1,561	1,880	1,740	1,916	9,894
5	304	670	664	764	753	679	557	682	5,073
6	209	956	823	807	852	866	780	643	5,936
7	128	1033	780	805	725	824	824	978	6,097
8	49	593	492	420	442	289	218	148	2,651
9	22	471	422	334	310	201	156	136	2,052
10	20	544	440	338	288	228	197	198	2,253
11	27	512	469	425	301	268	220	216	2,438
12	15	622	778	657	501	473	416	430	3,892
13		52	83	49	48	29	25	9	295
14		2	8		4				14
15				1			1		2
18							1		1
22								1	1

Data on the total number of dairy herds present in Ireland during each of these years were not available

**Table 2:** Summary statistics for herds that milk recorded during 2010

Parameter	Minimum	Maximum	25^th ^percentile	Median	75^th ^percentile
Average milk yield (L) per recording	2.7	49.1	17.7	21.8	25.5
Days in milk per recording	8	627	111	170	217
Number of cows per recording	10	568	42	59	82

**Figure 1:**
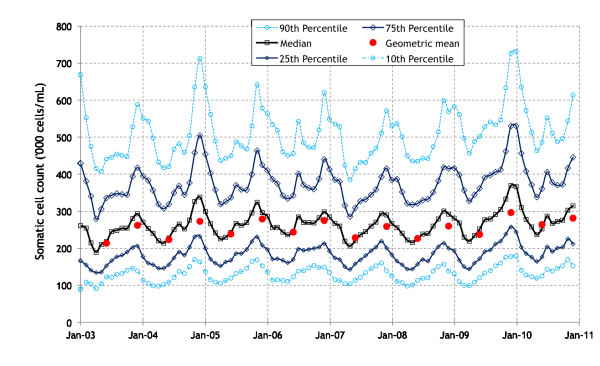
**The distribution (median, percentiles per month; geometric mean every 6 months) of unadjusted herd SCC results, based on SCC data from milk-recording Irish herds during 2003 to 2010**. If more than one valid milk recordings per herd was available for any month, only the first was used.

**Figure 2:**
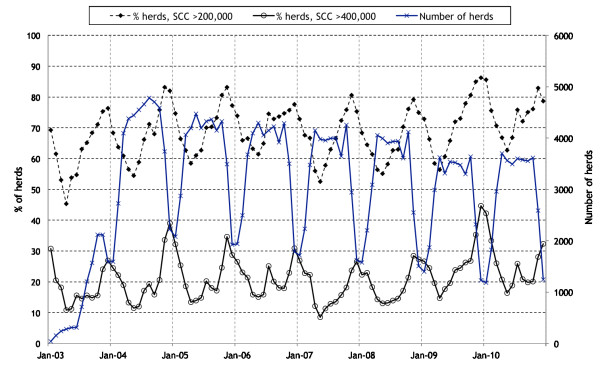
**The percentage of Irish dairy herds with a SCC result exceeding 200,000 and 400,000 cells/mL based on unadjusted SCC data from milk-recording herds, and the total number of herds milk recording, during 2003 to 2010**. If more than one valid milk recordings per herd was available for any month, only the first was used.

The distribution of unadjusted herd SCC during 2003 to 2010 is presented in Figure 1. A highly seasonal pattern is evident, with an increase in SCC (both median and range) between June and December each year. The percentage and number of herds during this period with an unadjusted herd SCC exceeding 200,000 and 400,000 cells/mL is presented in Figure 2. The percentage of herds exceeding 200,000 cells/mL varied from a low of 45% in April 2003 to a high of 86% in December 2009, and the percentage exceeding 400,000 cells/mL varied from a low of 9% in May 2007 to a high of 45% in December 2009. There was also a seasonal pattern in herd SCC results exceeding 200,000 and 400,000 cells/mL, except in 2003 (when milk recording data were sparse) (Figure 2).

**Figure 3:**
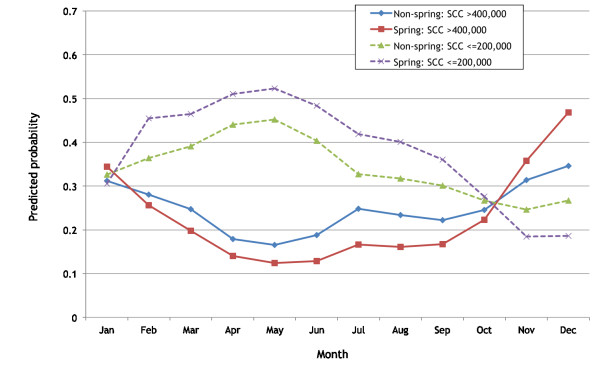
**The predicted probability of a herd either having a SCC result exceeding 400,000 cells/mL or < = 200,000 cells/mL based on the results from the final logistic GEE models for small herds (Q1) in 2004 comparing herds calving in Spring against all other herds**.

**Figure 4:**
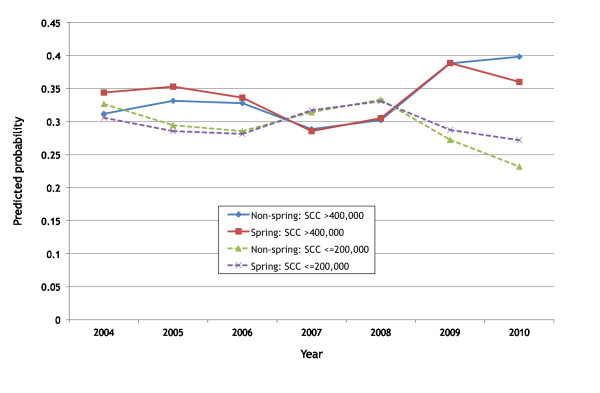
**The predicted probability of a herd either having a SCC result exceeding 400,000 cells/mL or < = 200,000 cells/mL based on the results from the final logistic GEE models for small herds (Q1) in January comparing herds calving in Spring against all other herds**.

The results from the final GEE models are shown in Tables 3 and 4. For ease of interpretation of the model the predicted probabilities by Spring calving, year and month are shown in Figures 3 and 4, in order to calculate the predicted probabilities the other variables were set to small herds in January (for the year calculations) and 2004 (for the month calculations).

**Table 3:** Logistic GEE model for the probability of a herd with an SCC > 400,000 cells/mL

Variable		*b*	**S. E**.	p-value	OR	95% CI	
Intercept		-0.79	0.04	< 0.001	0.45	0.42	0.49
Month	1	0.00	0.00	.	1.00	1.00	1.00
	2	-0.15	0.03	< 0.001	0.86	0.82	0.91
	3	-0.32	0.03	< 0.001	0.73	0.69	0.76
	4	-0.73	0.03	< 0.001	0.48	0.46	0.51
	5	-0.83	0.03	< 0.001	0.44	0.41	0.46
	6	-0.67	0.03	< 0.001	0.51	0.48	0.54
	7	-0.32	0.03	< 0.001	0.73	0.69	0.77
	8	-0.39	0.03	< 0.001	0.67	0.64	0.71
	9	-0.46	0.03	< 0.001	0.63	0.60	0.67
	10	-0.33	0.03	< 0.001	0.72	0.68	0.76
	11	0.01	0.03	0.735	1.01	0.96	1.06
	12	0.16	0.03	< 0.001	1.17	1.11	1.23
Year	2004	0.00	0.00	.	1.00	1.00	1.00
	2005	0.09	0.02	< 0.001	1.09	1.04	1.15
	2006	0.08	0.03	0.007	1.08	1.02	1.14
	2007	-0.11	0.03	< 0.001	0.90	0.84	0.95
	2008	-0.04	0.03	0.167	0.96	0.90	1.02
	2009	0.34	0.03	< 0.001	1.40	1.32	1.49
	2010	0.38	0.03	< 0.001	1.46	1.37	1.56
Herd size^a^	Q1	0.00	0.00	.	1.00	1.00	1.00
	Q2	-0.15	0.03	< 0.001	0.86	0.81	0.91
	Q3	-0.24	0.03	< 0.001	0.79	0.74	0.84
	Q4	-0.31	0.04	< 0.001	0.73	0.68	0.79
Spring Calving	Non-Spring	0.00	0.00	.	1.00	1.00	1.00
	Spring	0.15	0.07	0.049	1.16	1.00	1.34
Spring × Month^b^	Spring: 1	1	0.00	.	1.00	1.00	1.00
	Spring: 2	1	-0.27	< 0.001	0.76	0.66	0.88
	Spring: 3	1	-0.43	< 0.001	0.65	0.56	0.75
	Spring: 4	1	-0.44	< 0.001	0.64	0.56	0.74
	Spring: 5	1	-0.48	< 0.001	0.62	0.53	0.71
	Spring: 6	1	-0.60	< 0.001	0.55	0.48	0.63
	Spring: 7	1	-0.65	< 0.001	0.52	0.45	0.60
	Spring: 8	1	-0.61	< 0.001	0.54	0.47	0.62
	Spring: 9	1	-0.50	< 0.001	0.61	0.53	0.70
	Spring: 10	1	-0.28	< 0.001	0.76	0.66	0.87
	Spring: 11	1	0.05	0.478	1.05	0.91	1.21
	Spring: 12	1	0.36	< 0.001	1.43	1.23	1.68
Spring × Year^b^	Spring: 2004	0.00	0.00	.	1.00	1.00	1.00
	Spring: 2005	-0.05	0.05	0.310	0.95	0.86	1.05
	Spring: 2006	-0.11	0.05	0.037	0.90	0.81	0.99
	Spring: 2007	-0.16	0.06	0.004	0.85	0.76	0.95
	Spring: 2008	-0.14	0.06	0.015	0.87	0.78	0.97
	Spring: 2009	-0.14	0.06	0.010	0.87	0.78	0.97
	Spring: 2010	-0.31	0.06	< 0.001	0.73	0.66	0.82

^a ^Herd size groups were based on the quartile of the herd size within each year

^b ^Non-Spring herds were the reference category and set to 0

**Table 4:** Logistic GEE model for the probability of a herd with an SCC ≤ 200,000 cells/mL

Variable		*b*	**S. E**.	p-value	OR	95% CI	
Intercept		-0.72	0.04	< 0.001	0.49	0.45	0.52
Month	1	0.00	0.00	.	1.00	1.00	1.00
	2	0.17	0.03	< 0.001	1.18	1.12	1.24
	3	0.28	0.03	< 0.001	1.32	1.26	1.39
	4	0.48	0.03	< 0.001	1.62	1.54	1.71
	5	0.53	0.03	< 0.001	1.70	1.62	1.79
	6	0.33	0.03	< 0.001	1.39	1.32	1.47
	7	0.00	0.03	0.908	1.00	0.95	1.06
	8	-0.04	0.03	0.133	0.96	0.91	1.01
	9	-0.12	0.03	< 0.001	0.89	0.84	0.94
	10	-0.29	0.03	< 0.001	0.75	0.71	0.79
	11	-0.40	0.03	< 0.001	0.67	0.64	0.71
	12	-0.29	0.03	< 0.001	0.75	0.71	0.80
Year	2004	0.00	0.00	.	1.00	1.00	1.00
	2005	-0.15	0.02	< 0.001	0.86	0.82	0.90
	2006	-0.19	0.03	< 0.001	0.82	0.78	0.87
	2007	-0.06	0.03	0.036	0.94	0.89	1.00
	2008	0.03	0.03	0.284	1.03	0.97	1.09
	2009	-0.26	0.03	< 0.001	0.77	0.73	0.82
	2010	-0.48	0.03	< 0.001	0.62	0.58	0.66
Herd size^a^	Q1	0.00	0.00	.	1.00	1.00	1.00
	Q2	-0.10	0.03	0.001	0.91	0.86	0.96
	Q3	-0.15	0.03	< 0.001	0.86	0.81	0.91
	Q4	-0.36	0.03	< 0.001	0.70	0.65	0.75
Spring calving	Non-Spring	0.00	0.00	.	1.00	1.00	1.00
	Spring	-0.10	0.08	0.211	0.91	0.78	1.06
Spring × Month^b^	Spring: 1	0.00	0.00	.	1.00	1.00	1.00
	Spring: 2	0.47	0.07	< 0.001	1.61	1.39	1.86
	Spring: 3	0.40	0.07	< 0.001	1.49	1.30	1.72
	Spring: 4	0.38	0.07	< 0.001	1.46	1.27	1.68
	Spring: 5	0.38	0.07	< 0.001	1.46	1.27	1.68
	Spring: 6	0.42	0.07	< 0.001	1.53	1.33	1.76
	Spring: 7	0.49	0.07	< 0.001	1.63	1.41	1.88
	Spring: 8	0.46	0.07	< 0.001	1.58	1.37	1.83
	Spring: 9	0.36	0.07	< 0.001	1.44	1.24	1.66
	Spring: 10	0.14	0.07	0.056	1.15	1.00	1.33
	Spring: 11	-0.27	0.08	0.001	0.76	0.66	0.89
	Spring: 12	-0.37	0.09	< 0.001	0.69	0.58	0.83
Spring × Year^b^	Spring: 2004	0.00	0.00	.	1.00	1.00	1.00
	Spring: 2005	0.05	0.04	0.221	1.05	0.97	1.15
	Spring: 2006	0.07	0.05	0.107	1.08	0.98	1.18
	Spring: 2007	0.11	0.05	0.017	1.12	1.02	1.23
	Spring: 2008	0.08	0.05	0.076	1.09	0.99	1.20
	Spring: 2009	0.17	0.05	0.001	1.19	1.08	1.31
	Spring: 2010	0.31	0.05	< 0.001	1.36	1.23	1.51

^a ^Herd size groups were based on the quartile of the herd size within each year

^b ^Non-Spring herds were the reference category and set to 0

The odds of a high SCC (> 400,000 cells/mL) was highest in December, with the lowest probability in May (Table 3 Figure 3). Spring herds had a lower probability of a high SCC during February to October compared to non-Spring herds but a higher probability between November to January. The odds of a high SCC were greater in 2005, 2006, 2009 and 2010 but less in 2007 and 2008 compared to 2004. Spring herds had a higher probability of a high SCC in 2004 and 2005 compared to non-Spring herds and they had a lower probability in 2010 compared to non-Spring herds (Figure 4). Smaller herds had higher odds of having a high SCC compared to larger herds. Pearson residuals were examined using a half-normal plot with simulated envelope. There were no indications that the model was inappropriate.

The odds of a low SCC (≤ 200,000 cells/mL) was highest from February to June, with the lowest probability in November (Table 4). Spring herds had a higher probability of a low SCC in every month except November, December and January. The odds of a low SCC were greater in 2004, 2007 and 2008. Spring herds had a higher probability of a low SCC in 2004, 2009 and 2010. Smaller herds had higher odds of having a low SCC compared to larger herds.

Table 5 shows the proportion of herd recordings that were: ≤ 200,000, > 200,001-400,000, and > 400,000 cells/mL, by herd size group. Note that the herd size groupings varied with the respective yearly herd size distribution, with Q1 being the quarter of smallest herds in the population, and Q3 being the quarter of largest herds in the population. The smallest herds tended to have more recordings in the extremes of SCC readings (22% were > 400,000, 35% were ≤ 200,000 cells/mL), whilst the largest herds tended to have a higher proportion of recordings in the middle SCC range (55% of recordings were > 200,001-400,000 cells/mL for larger herds).

**Table 5:** Proportion of herd recordings within each grouping of SCC, by herd size group

	SCC		
	
Herd size	< = 200	201-400	> 400
Q1	35.52	42.35	22.12
Q2	33.06	46.99	19.96
Q3	31.38	49.47	19.15
Q4	26.50	54.74	18.76

### Antibiotic sales

**Figure 5:**
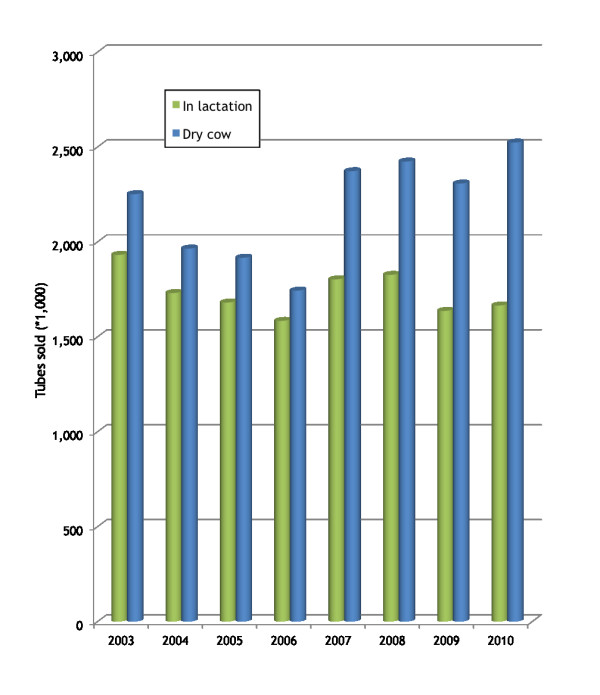
**The number of intramammary antibiotic tubes sold annually in Ireland during 2003-2010, by product type (in-lactation and dry cow use)**.

The number of intramammary tubes sold annually in Ireland during 2003-2010, by product type (in-lactation and dry cow use) is presented in Figure 5. Table 6 presents the quantity of active substance (kg) in intramammary tubes sold annually in Ireland during 2003-2010, by product type (in-lactation and dry cow use) and antibiotic group. Almost 2 tonnes of active substance (1,677 kg of active substance for in-lactation and dry cow use, data represents 85% of sales) was sold for use in 2010, from a range of antibiotic groups (for in-lactation usage: aminoglycocides, broad spectrum penicillins and 1^st ^generation cephalosporins [from most frequent by weight]; for DCT: penicillinase-resistant narrow spectrum penicillins, 1^st ^generation cephalosporins and β-lactamase sensitive, narrow spectrum penicillins for DCT).

**Table 6:** The quantity of active substance (kg) in intramammary tubes sold annually in Ireland during 2003-2010, by product type (in-lactation [Lact.] and dry cow [DC] use) and antibiotic group

	2003		2004		2005		2006		2007		2008		2009		2010	
	
	**Lact**.	DC	**Lact**.	DC	**Lact**.	DC	**Lact**.	DC	**Lact**.	DC	**Lact**.	DC	**Lact**.	DC	**Lact**.	DC
*Aminocoumarin*	56.6	0.2	50.5	-	45.6	-	40.8	-	48.8	-	42.9	-	41.4	-	51.9	1.6
*Aminoglycosides*	269.6	53.8	239.8	43.8	227.6	38.6	206.7	33.7	230.2	32.8	259.9	90.1	218.0	66.5	170.0	38.8
*Cephalosporins*																
1^st ^generation	85.6	147.5	80.3	156.4	74.2	165.1	71.2	171.5	68.5	212.6	77.6	270.6	74.3	274.0	89.1	296.5
3^rd ^generation	2.0	-	2.1	-	3.6	-	3.7	-	4.0	-	4.5	-	4.3	1.1	8.3	8.1
4^th ^generation	1.0	-	1.3	-	1.8	-	1.4	-	2.2	-	2.5	-	1.8	-	2.0	-
*Lincosamides*	10.5	-	5.4	-	4.6	-	3.6	-	3.4	-	4.7	-	3.4	-	3.9	-
*Macrolides*	0.9	-	0.6	-	0.2	-	-	-	-	-	-	-	-	-	-	-
*Pencillins*																
Narrow spectrum																
β-lactamase sensitive	120.3	204.3	104.9	163.3	100.3	145.1	89.7	114.8	101.7	128.0	107.3	137.5	91.2	137.2	55.5	167.6
Penicillinase-resistant	3.1	656.4	2.7	532.6	3.1	512.6	3.8	448.0	2.3	686.7	1.7	635.3	1.6	528.4	2.9	511.5
Moderate spectrum	1.1	161.8	1.0	147.2	1.2	144.8	1.4	133.0	0.9	204.9	0.6	144.7	0.6	114.0	1.1	139.2
Broad spectrum	97.7	-	87.7	-	89.4	-	92.2	-	105.5	-	102.0	-	101.2	-	124.7	-
*Sulphonamides*	1.7	-	1.5	-	1.2	-	0.6	-	17.1	-	5.0	-	3.1	-	3.7	-
*Tetracyclines*	3.6	-	3.6	-	4.2	-	3.9	-	4.6	-	11.9	-	4.4	-	-	-

Total	653.6	1,224.0	581.5	1,043.2	556.8	1,006.3	519.1	900.9	589.2	1,265.0	620.6	1,278.2	545.4	1,121.2	513.3	1,163.3

In 2010, the estimated DCT coverage was approximately 92.7% (Table 7). The estimated incidence of clinical mastitis was 54.0 cases per 100 cow-years at risk, assuming 4 tubes per treatment regime, one affected quarter per cow, tubes restricted to clinical cases only and 100% of treated cases considered new cases (Table 8). With differing assumptions, this estimate varied between 25.8 and 77.0 cases per 100 cow-years at risk.

**Table 7:** Estimated coverage of dry cow therapy (DCT) in Ireland during 2010, based on DCT sales collated by GfK Kynetec

			Explanation, assumptions
i.	Approximate number of dairy cows (2010)	1,000,000	Includes all milking cows (lactation 1+)
ii.	Approximate number eligible for DCT	800,000	i. minus (cows in final lactation), assume 5 lactations. DCT is not administered to yet-to-calve heifers
iii.	Number of dry cow tubes sold (2010)	2,522,500	As collated by GfK Kynetec
iv.	Total number of dry cow tubes sold	2,967,647	Assuming iii. represents 85% of all DCT sales
v.	Estimated DCT coverage	92.7%^a^	Assuming a single tube per quarter, four quarters per cow

a. This figure would be 98.5% or 87.6% if GfK Kynetec data represents 80% or 90% of national DCT sales, respectively

**Table 8:** Estimated incidence of clinical mastitis (cases per 100 cows per lactation) in Ireland during 2010, using three different scenarios (with differing numbers of tubes per treatment regime) and based on DCT sales collated by GfK Kynetec

			Explanation, assumptions
i.	Approximate number of dairy cows (2010)	1,000,000	Includes all milking cows, assuming an average 5 lactations/cow
ii.	Number of cows at risk of clinical mastitis	1,000,000	Equals i. (all lactating cows are at risk of clinical mastitis)
iii.	Days at risk per cow	335	Assumes 305 day lactation, 60 day dry period and cows are at risk from 30 days prior to calving until drying off^a^
iv.	Days after treatment before a subsequent treatment is considered a new case	8	As recommended^a^
v.	Number of in-lactation tubes sold (2010)	1,664,066	As collated by GfK Kynetec
vi.	Total number of in-lactation tubes sold	1,957,725	Assuming v. represents 85% of all DCT sales

Scenario 1: 3 tubes per treatment regime			

1.i	Total number of treated clinical cases	652,575	Assuming 3 tubes per treatment regime, one affected quarter per cow, tubes restricted to clinical cases only
1.ii	Total number of new cases	652,575	100% of treated cases considered new cases
1.iii	Number of clinical cases per 100 cow-years at risk	72.4^b, c^	

Scenario 2: 4 tubes per treatment regime			

2.i	Total number of treated clinical cases	489,431	Assuming 4 tubes per treatment regime, one affected quarter per cow, tubes restricted to clinical cases only
2.ii	Total number of new cases	489,431	100% of treated cases considered new cases
2.iii	Number of clinical cases per 100 cow-years at risk	54.0^b, c^	

Scenario 3: 5 tubes per treatment regime			

3.i	Total number of treated clinical cases	391,545	Assuming 5 tubes per treatment regime, one affected quarter per cow, tubes restricted to clinical cases only
3.ii	Total number of new cases	391,545	100% of treated cases considered new cases
3.iii	Number of clinical cases per 100 cow-years at risk	43.1^b, c^	

a. Based on recommendations of the International Dairy Federation [21]

b. Without changing any other assumptions, if GfK Kynetec data represents 80% or 90% of national DCT sales, the incidence of clinical cases would range from 77.0 to 68.3 in scenario 1, 57.5 to 51.0 in scenario 2 and 45.8 to 40.7 in scenario 3, respectively

c. Without changing any other assumptions, if the percentage of treated cases considered new cases were 80% or 60%, the incidence of clinical cases per 100 cow-years at risk would be 57.7 or 43.1 for scenario 1, 43.1 or 32.3 for scenario 2 and 34.4 or 25.8 for scenario 3, respectively

### Animal health assessment

During 2003 to 2009, there were no known cases where a dairy cert. had not been issued.

## Discussion

Objective measurement is critical to improvement. For this reason, it is important that Ireland has an objective understanding of current levels of udder health, to facilitate benchmarking and improvement into the future. This is particularly important, given the recent establishment of *CellCheck*, a national programme to support SCC improvement throughout Ireland, as discussed later.

Udder health is a concern on a number of Irish dairy farms. As highlighted in Figures 1 and 2, a substantial number of milk-recorded herds had an elevated unadjusted herd SCC (exceeding 400,000 cells/mL) during the study period (2003 to 2010). High SCC results were present throughout the year, but more marked towards the start and end of each milking season. Note that milk supply in Ireland is highly seasonal, as reflected in Figure 2 (specifically, the number of herds that milk recorded during the years of interest). Compared with 2004, the odds of a herd having an SCC > 400,000 cells/mL were greater in 2005, 2006, 2009 and 2010, but less in 2007 and 2008. Further, herds in 2009 and 2010 had a 30% higher odds of having an SCC > 400,000 cells/mL compared to herds in 2004. Smaller herds tended to have more extreme SCC recordings (very low and very high) compared with larger herds. These results confirm earlier concerns from previous Irish studies [17], where an analysis of bulk milk tank (payment) data during 1994 to 2004, inclusive, was conducted. In this study [17], a year-on-year increase in SCC from 2000 was noted. Bulk milk tank data provides insight into the quality of milk at the point of dispatch from a farm, noting that farmers may withhold cows with high SCC from the bulk tank. In contrast, milk recording is generally conducted on all cows lactating at the time of recording, providing a more accurate reflection of on-farm udder health. Based on the results of an earlier Irish study, milk recording herds have lower cell counts than the national average [18].

In this paper, we have used intramammary antibiotic sales data to estimate both DCT coverage and the incidence of clinical mastitis in Ireland during 2010. Although no alternative data are currently available, these estimates must be interpreted with caution. Each is underpinned by both estimates (of lactating cow numbers, of total sales) and assumptions. For this reason, we have conducted a sensitivity analysis with each calculation, highlighting the impact of changes in these assumptions on DCT coverage and clinical mastitis incidence. We estimate that the GfK Kynetec sales data represents approximately 85% of total sales, guided by those closely involved in this industry. Manufacturers generally recommend 3 tubes per treatment regime, however, Blowey and Edmondson [22] suggest the average usage in the UK is closer to 4-5 tubes per cow. In Ireland, some farmers may use fewer tubes per treatment regime, to minimise treatment costs. In our calculations, we consider a range in the number of tubes per treatment regime. No account was taken of repeat cases as such data were not available, which will lead to some inflation of estimates of clinical case incidence. In prospective studies, repeat cases (defined as one that recurs in the same quarter more than one week after the last day of treatment [23]) can be avoided by discarding any cows with a case of clinical mastitis in the previous four weeks [24].

Based on the data presented (Table 8), blanket DCT is common practice throughout Ireland, with estimated coverage of 92.7%. Blanket DCT is generally recommended as part of a comprehensive on-farm mastitis control plan, except on farms where mastitis is under very good control (comprehensive individual milk recording, clear microbiological picture, few cows with peak SCC over 250,000 cells/ml etc.), with a low prevalence of infected cows at drying off, and a low rate of new intra-mammary infections in the dry period.

The study results reflect a relatively high incidence of clinical mastitis in Ireland (54.0 clinical cases per 100 cow-years at risk) using methods recommended by the International Dairy Federation [21]. This estimate is very dependent on the assumptions used, as outlined in Table 8. Further, we assume that tubes were restricted to clinical cases. The incidence of clinical mastitis in dairy cattle varies widely, due to differences in a range of factors including climate, level of production and management [24]. In the UK, a mean incidence rate of clinical mastitis of between 47 and 71 cases per 100 cows per year was identified [24], which is higher than reported previously [25]. In the Netherlands, Miltenburg et al. [26] calculated an incidence rate of 12.7 quarter cases per 100 cows per year (repeat cases considered new if > 14 days apart). In Australia, professional input is recommended within the *Countdown Downunder *programme when clinical cases exceeds 5 per 100 cows during the first month of lactation or 2 per 100 cows in each month subsequently (a maximum of 23 cases per 100 cows per year, assuming a 10 month lactation) [27].

There is widespread concern about the use of unnecessary and excessive use of antibiotics in farm animals [28], with significant implications for human health, particularly in terms of antibiotic resistance [29]. There is particular concern with the non-therapeutic use of antibiotics, primarily in the form of feed additives, particularly during poultry and pig production [29]. The impact of antibiotic use in dairy cows was recently reviewed [30]. Based on this review, there is currently no evidence of 'widespread, emerging resistance among mastitis pathogens to antibacterial drugs'. However, 'the use of antibiotics in food-producing animals does contribute to increased antimicrobial resistance', highlighting the need for 'prudent use of antibiotics in the dairy industry'.

In this paper, we infer characteristics of on-farm intramammary antibiotic usage based on sales data collated by GfK Kynetec. In most countries, including Ireland, data on antibiotic usage is generally not available [31]. Several published reports of on-farm intramammary antibiotic usage are available, for example from Switzerland [32] and the USA [33,34], however, these have relied on farm surveys. In a recent review [31], it was recommended that national monitoring programmes be established of antimicrobial usage in food animals, using data from a range of sources including sales data, with these data being reported annually, including the total amounts of each compound and kilograms of each active ingredient. Similar suggestions were made in a recent communication from the European Commission on antimicrobial resistance, with key recommendations including:

• Strengthening the regulatory framework on veterinary medicines, seeking prudent use of antimicrobials in veterinary medicine *(action no. 2)*;

• Introducing the new EU Animal Health Law, which will focus on prevention of diseases, reducing the use of antibiotics and replacing current Animal Health provisions based on disease control *(action no. 5)*; and

• Strengthening surveillance systems on antimicrobial resistance and antimicrobial consumption in animal medicine, including promotion and extension of the European Surveillance of Veterinary Antimicrobial Consumption (ESVAC). to obtain harmonized data on the usage per animal species and production categories *(action no. 10) *[35].

The use of intramammary antibiotics in the Irish dairy industry is regulated under both Irish and EU law. The GfK Kynetec sales data provides a valuable insight into on-farm usage. Nonetheless, we acknowledge that our usage estimates must be interpreted with care. During 2003-2010, there appears to have been substantial usage of intramammary antibiotics in the Irish dairy herd. Unfortunately, few international comparisons are available. Some interpretation of this usage is possible, incorporating this and other data. As background, it is important to be aware that *Staphylococcus aureus *is the most frequently isolated mastitis pathogen in Ireland, based on culture results from milk sample submissions to Regional Veterinary Laboratories during 2005-2010 [36-39] and a study of 300 bulk milk tank samples collected during 2006 [19]. In 2009, *S. aureus *was isolated from 43.6% of 5,004 milk sample submissions [39]. Given this microbiological picture, the common practice of blanket DCT in Ireland (as highlighted previously) is consistent with rational antibiotic usage. In contrast, these data suggest a high rate of antibiotic usage during lactation, most likely in response to clinical cases of mastitis. However, the efficacy of mastitis therapy for chronic *Staphylococcus aureus *infection during lactation is extremely low, leading to very low cure rates following treatment [30]. Therefore, there may be an over-reliance on antibiotics in the dairy industry in Ireland in situations where the efficacy of treatment is low. Based on the data available, we do not observe any substantive impact of legislative change (in January 2008) on intramammary antibiotic sales in Ireland. More work is needed nationally to both evaluate and monitor intramammary antibiotic usage, including comparison with international models of best practice.

The dairy cert. (or Animal Health Inspection of Dairy Cows) was introduced by the Irish government as a means to certify compliance with animal health requirements. It is of surprise that all herds have been found in compliance in each of the years under study, particularly given the scope of the above-mentioned udder health concerns. It should be noted that the wording of the dairy cert. is ambiguous or otherwise open to interpretation, which may limit its usefulness. As one example, veterinarians are asked to certify that farms are compliant with the requirement of *'... a general state of health that is not impaired by.. a recognisable inflammation of the udder.' *This could be interpreted to include any herd with mastitic cows at the time of inspection, a requirement that would be difficult to achieve in any large dairy farm internationally. A critical assessment of the usefulness of the dairy cert. would seem justified.

In this study, we used three different data sources to gain an insight into udder health on Irish dairy farms. This approach was needed, as no national data are available providing a complete picture of udder health in Ireland. Each of these data sources has its strengths and limitations. The milk recording data are robust, providing an accurate picture of udder health among the cows under test. By definition, however, it only relates to milk recording herds; in 2008, milk recording was conducted on 32% (6,400 of an estimated 20,000) of Ireland's dairy herds. Further, milk recording only became widely established in 2004, hence the scarcity of data prior to this. The GfK Kynetec dataset was based on actual sales, with account being taken of any product returns. These (sales) data are comprehensive, including most, but not all, sales of these products in Ireland. In this study, we have used these sales data to estimate on-farm usage, providing a detailed outline of all relevant assumptions in Tables 7 and 8. The animal health assessment data is collated at the level of the cooperative, with any aberrations reported centrally to DAFM.

Animal Health Ireland (AHI) is Ireland's national coordinating body in non-regulatory animal health [40,41]. AHI recently established *CellCheck *as the national programme to support SCC improvement throughout Ireland. The programme is supported by all sectors in the dairy industry (including farmers, cooperatives, processors and national coordinating groups), by government and by all relevant service providers (veterinarians, Teagasc [the Irish Agriculture and Food Development Authority] and other milk quality advisors, milking machine technicians), noting that industry standards and economic signals will play a key role in motivating continuous improvement. The *CellCheck *programme is multifaceted, but includes the development of detailed resource material suitable for farmers and their service-providers, and associated training programmes. AHI is working closely with ICBF to develop farm-based and national tools to monitor and analyse progress, and with universities and Teagasc to conduct relevant economic and sociological research. The programme is an adaptation of *Countdown Downunder *[27,42], a highly successful programme to resolve SCC concerns in Australia.

The current work provides insights into udder health and intramammary antibiotic usage in Ireland during the last 8 years. This information is critical, providing an objective benchmark for improvement. Similar work is needed into the future. We are also contributing to additional research in support of *CellCheck*. In collaboration with relevant stakeholders, from industry, government and service providers, we are investigating challenges (political, economic, social and technological) and opportunities to improving udder health in Ireland. Work is also ongoing to investigate the effect of data adjustment, as outlined in EU legislation, on herd eligibility to supply raw milk for human consumption.

## Competing interests

None of the authors has any financial or personal relationships that could inappropriately influence or bias the content of the paper.

## Authors' contributions

SM coordinated the study, assembled the data and led in the writing of the manuscript. TC analysed the data and assisted in the writing of the manuscript. LOG provided expert advice on methods to assess milk quality and mastitis control. All authors read and approved the final manuscript.

## Supplementary Material

Additional file 1 **An objective assessment of milk quality in Ireland during 2003-2010 **[43-59]. Click here for file
