# Supplementary material for: Insights into udder health and intramammary antibiotic usage on Irish dairy farms during 2003-2010
Source: Ir Vet J. 2012 Mar 28;65(1):7. doi: 10.1186/2046-0481-65-7 (PMC3376034; doi:10.1186/2046-0481-65-7)
Supplement: Additional file 1 — An objective assessment of milk quality in Ireland during 2003-2010 [43-59]. [file 2046-0481-65-7-S1.DOC]

**Insights into udder health and intramammary antibiotic usage on Irish dairy farms during 2003–2010**

**Additional file 1**

a. Relevant legislation

Prior to 2005, milk quality standards were directed by the EU Milk Directive (92/46/EEC) [43] which required all milk producers to conform to minimum health rules for the production and placing of raw milk, heat treated milk and milk-based products on the market. Since January 2006, the ‘hygiene package’ (encompassed in Regulations 852/2004 [44], 853/2004 [45], 854/2004 [46], 882/2004 [47], 2073/2005 [48] and Directive 2002/99 [49]) has formed the basis for EU food safety legislation. Together with Regulation 178/2002 (general food law) [50], this package has sought to provide a holistic approach to safety assurance across the entire food chain from primary production inputs, through to point of supply to the final consumer. It emphasises the use of risk-based (rather than prescriptive) approaches to food safety, and HACCP (hazard analysis and critical control points) principles are now mandatory at all stages of the food chain, apart from primary production. Under this package, all food business operators (FBOs, including primary producers) will bear primary responsibility for food safety assurance at their level of the food chain. It is the role of the Competent Authorities (such as the Department of Agriculture, Food and the Marine, DAFM, and the Food Safety Authority of Ireland, FSAI) to verify compliance by the FBO [51]. As required under EU Regulation 882/2004 [47], a national control plan for Ireland for the period 2007 to 2011 was developed, presenting general information on the structure and organisation of the systems of controls for food, feed, animal health and animal welfare in Ireland [52].

Prior to 2008, intramammary antibiotic products were classified as licensed merchant (LM) products, and as such were freely available from a range of outlets throughout Ireland, without prescription. In compliance with EU Directive 2004/28/EC [53], these products were upgraded to prescription-only medicines (POMs) on 1 January 2008. In contrast to all other POMs, under Irish legislation (S.I. 786 of 2007) [54] these products may be prescribed by a registered veterinary practitioner without a requirement for at least 12 monthly on-farm visits, provided the herd is part of a programme, as defined in this legislation.

As required under EU Regulation 853/2004 [45], raw milk must comply with microbiological criteria and standards for plate count and somatic cell count. These include requirements that raw milk has a total bacterial count not exceeding 100,000 cells/mL (based on a rolling geometric average over a two month period, with at least two samples per month) and a somatic cell count not exceeding 400,000 cells/mL (based on a rolling geometric average over a three month period, with at least one sample per month). EU Regulation 854/2004 [46] states that, with respect to somatic cell counts, once the three month geometric mean somatic cell count reaches 400,000 cells/mL, the competent authority must issue a warning. The supplier then has three months in which to reduce his/her three month geometric mean to below the 400,000 cells/mL limit.

There are no specific legislative requirements for thermoduric bacteria (by definition, bacteria that survive pasteurisation). As provided in EU Decision 96/360/EC [55], Ireland applies an adjusted calculation method (weighting the SCC results of November to February) to account for seasonal variations in production levels, and where excesses ‘have a physiological basis and cannot be ascribed to a disease of the udder’. The maximum residue limits for veterinary medicinal products, including antibiotics, in milk are outlined in EU Regulation 2377/90 [56].

b. Legislative enforcement

DAFM is the designated competent authority for enforcement of milk quality legislation relevant to on-farm production. DAFM is responsible for the registration and ongoing monitoring, inspection and auditing. Inspections are conducted either by DAFM’s agricultural inspectorate (for farms supplying manufacturing milk) or by the local authorities' public health protection service (for farms supplying drinking milk) [52]. Each farm is also inspected periodically by a veterinarian to certify compliance with animal health requirements (the Animal Health Inspection of Dairy Cows or ‘dairy cert.’). On the date of inspection, all cows (milking and intended for milking) must:

- Not show any visible evidence of infectious diseases communicable to human beings through milk;
- Have a general state of health that is not impaired by any visible disorder and which are not suffering from any infection of the genital tract with discharge, or enteritis with a scour and fever, or a recognisable inflammation of the udder; and
- Not show any udder wound likely to affect the milk.

In consultation with the FSAI, DAFM’s state veterinary service is responsible for the design and implementation of a national milk residue monitoring plan, as required under EU Directive 96/23/EC [57]. A broad range of residue testing is conducted, including those classified as B1 (antibacterial substances: antibiotics [penicillins, tetracyclines, quinolones, aminoglycosides, macrolides] and sulphonamides [sulfadiazine, sulfadimethoxine, sulfamethazine, sulfathiazol]). The plan is conducted using targeted sampling. Samples are initially screened using microbial inhibition tests; chromatographic methods [58] are used to identify and confirm the identity and quantity of antibiotic present.

Milk processors are responsible for checking the quality of milk at the time of initial processing. Each processor collects detailed data on bulk milk SCCs and TBCs (including thermoduric bacteria), and provides monthly summary data to DAFM of the number of suppliers barred from supplying milk due to excessive SCC and TBC counts. The processor is responsible to ensure that a valid dairy cert. is available from each of its farm suppliers. As outlined in Irish legislation (S.I. 143 of 2007) [59], processers are required to implement self-monitoring residue plans (‘self-checks’). Each processor is required to immediately notify DAFM in the event of a positive residue sample.

c. Legislative compliance

The Food and Veterinary Office (FVO) has responsibility, both within the European Union and in third countries exporting to the EU, for checking compliance with the requirements of EU food safety and quality, veterinary and plant health legislation. During 2003 to 2010, several FVO inspections were conducted focusing on compliance to relevant legislation, including:

- The control of residues in live animals and animal products (inspection number 9049/2003, October 2003);
- Food hygiene - food of animal origin (8166/2006, April 2006);
- Babyfood and infant formulae (7148/2007, September 2007); and
- General audit during February to November 2008 (8724/2008).
